# The Hidden Curriculum: Exposing the Unintended Lessons of Medical Education

**DOI:** 10.7759/cureus.845

**Published:** 2016-10-25

**Authors:** Laura Hopkins, Lana Saciragic, Joanna Kim, Glenn Posner

**Affiliations:** 1 Department of Gynecologic Oncology, The Ottawa Hospital; 2 Department of Gynecologic Oncology, Tom Baker Cancer Centre, Calgary; 3 Faculty of Medicine, University of Ottawa; 4 Department of Innovation in Medical Education, University of Ottawa

**Keywords:** hidden curriculum, clerkship, medical education, ob/gyn

## Abstract

Introduction: The hidden curriculum is a set of ethical, moral, and value-based teachings communicated to doctors-in-training, providing a basis for their future interactions with patients, peers, and colleagues. The aim of our study is to introduce the concept of the hidden curriculum to a cohort of third-year medical students and to subsequently evaluate their understanding. In particular, we sought to measure and benchmark the degree of hidden curriculum recognition within a Canadian medical education context. With the help of student feedback, we elicited ideas for future directions.

Methods: One hundred and fifty-four third-year medical students completing their obstetrics and gynaecology core clinical rotation attended a workshop on the hidden curriculum. Students completed two sets of evaluations; a voluntary anonymous pre- and post-workshop questionnaire evaluating their knowledge and opinions regarding the hidden curriculum, and a mandatory workshop evaluation. Answers to pre- and post-workshop questionnaires were compared using Mann-Whitney U test, and thematic analysis was used to code the students’ comments to identify common themes.

Results: A standardized workshop on the hidden curriculum significantly improved students’ understanding and highlighted the importance of the hidden curriculum. Voluntary student comments (N = 108) were categorized according to five themes:  1) Students who were not sensitized to the hidden curriculum (8; 7.4%); 2) students who were sensitized but unaware of the hidden curriculum (12; 11.1%); 3) students who were sensitized and aware of the hidden curriculum (34; 31.5%); 4) comments on teaching methodologies/environment (43; 39.8%); and 5) suggestions for enhancement (11; 10.2%).

Conclusions: A simple, cost-effective intervention, such as a workshop, can effectively assess and address the hidden curriculum. Many students are highly sensitized to and are aware of the positive and negative effects of role modeling on their development.  The students are calling for similar interventions to be directed at the postgraduate and faculty level.

## Introduction

Dr. Sally Mahood has described the hidden curriculum as, “more than simple transmission of knowledge and skills, it is also a socialization process…The hidden curriculum consists of what is implicitly taught by example day to day, not the explicit teaching of lectures, grand rounds, and seminars” [1]. In essence, it is a set of ethical, moral, and value-based teachings communicated, often in a non-explicit manner, to doctors-in-training. It is a foundation for communication and influences future interactions with patients, peers, and colleagues. The hidden curriculum can be a negative empathy-disabling curriculum, and its effect upon trainees is felt to be counter to the explicitly stated curriculum goals. The Association of Faculties of Medicine in Canada (AFMC) released a document in 2010, “The Future of Medical Education in Canada”, which explicitly recommends that the hidden curriculum be introduced into undergraduate education [2].

What is the importance of the hidden curriculum? First, it is critical to professional and emotional growth [3-5]. Secondly, it may have a direct benefit on patient care, as it has been demonstrated that patients engage in a more meaningful patient-physician relationship if they trust their care provider [6]. Thirdly, recognition and structured reflection on the hidden curriculum may provide resilience to burnout and, in turn, increase the productivity of the medical workforce [7]. In fact, the significance and implications of the hidden curriculum are shown to transcend the undergraduate level to include students, residents, and staff physicians [8]. Obstetrics and gynaecology is a broad and situationally intense field with many ethical considerations and, therefore, addressing the hidden curriculum within this rotation is relevant, appropriate, and necessary.

The hidden curriculum has become an integrated and mandatory part of undergraduate medical education in North America for several years [2, 9-10]. Thus far, only two Canadian medical schools have published their centre-specific interventions addressing the hidden curriculum [9, 11]. An online survey completed by the Association of Academic Professionals in Obstetrics and Gynecology (APOG) undergraduate rotation directors shows that the hidden curriculum is addressed in 9/17 (53%) medical schools in Canada. Despite these undertakings, to our knowledge, there are no studies specifically evaluating the effects of implementation of hidden curriculum training in medical education.

The aim of our study is to assess and address the hidden curriculum within a cohort of third-year medical students and subsequently to evaluate their understanding. In particular, we wish to measure and benchmark the degree of hidden curriculum recognition within a Canadian medical education context and, with the help of student feedback, elicit ideas for future directions.

As this workshop, including its evaluation, was a part of the mandatory curriculum, ethics board approval was not required.

## Materials and methods

One hundred and fifty-four third-year medical students at the University of Ottawa participated in this mixed methods study during the 2013-2014 academic year. These students had been exposed to the concept of the hidden curriculum in their pre-clerkship through a longitudinal ePortfolio platform. The students attended a mandatory, standardized workshop on the hidden curriculum during the first week of their six-week core clinical rotation in Obstetrics and Gynaecology. The Obstetrics and Gynaecology Undergraduate Rotation Director developed and facilitated all workshops. In total, there were eight small group sessions with 19-22 students each (N = 154). During the session, the students were provided an overview of the concept of the hidden curriculum, and then the class was divided into four smaller groups to facilitate structured case-based review and discussion. Students were subsequently encouraged to share their personal clinical experiences with the larger group for facilitated and supported discussion. In keeping with the work of Cohn, et al. [12], a process of self-reflection is used, as a means of providing the student with some takeaway skills for processing messages contained in the hidden curriculum. 

**Figure 1:**
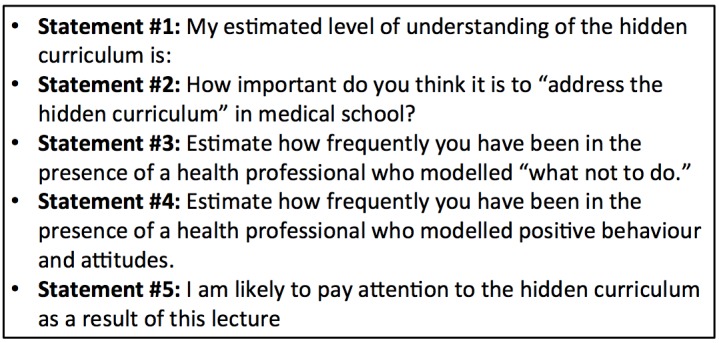
Pre and post-workshop questionnaire statements.

The students completed a voluntary anonymous pre- and post-workshop questionnaire, assessing their knowledge, experience, and attitudes regarding the hidden curriculum. The pre-workshop questionnaire presented four statements (Figure 1), and students were asked to rank their answers on a 10-point Likert scale. In the post-workshop questionnaire, students were presented with a fifth statement: “I am likely to pay attention to the hidden curriculum as a result of this lecture.” We grouped these responses into three distinct categories, based on how likely the students were to pay attention to the hidden curriculum in the future: “low" (grades 0-3), “moderate" (grades 4-7), and “high" (grades 8-10).

At the end of the session, the students completed a mandatory online course evaluation, as per the standard accepted template at the University of Ottawa. For the qualitative portion of the study, descriptive statistics were used to analyze the data, and the mean, median, and standard deviations for each question were calculated. Pre and post-workshop means were compared using the Mann-Whitney U test.

For the quantitative portion of the study, the analysis team (LH, LS, and JK) evaluated the written comments using inductive, thematic analysis to discover emerging patterns and themes [13]. The team systematically examined all data, through three cycles of independent, then group analysis. One cycle was used to achieve each of the following goals: to identify emergent trends, develop thematic categories, and then refine them. We explored the data further to identify any trends in responses that might be reflective of the duration of prior clerkship experience. Team meetings were held to resolve discrepancies, review deviant cases [14], and build a common understanding of the data. Triangulation was achieved through the collaboration of three researchers and the use of the dataset [15].

As this workshop, including its evaluation, was a part of the mandatory curriculum, ethics board approval was not required and participant consent was waived.

## Results

A total of 154 third year medical students participated in the hidden curriculum workshop. Sixty-three (41%) were males and 91 (59%) females. The age range was 23 – 39 years old, with the mean age being 26. 

### Voluntary questionnaire

**Figure 2:**
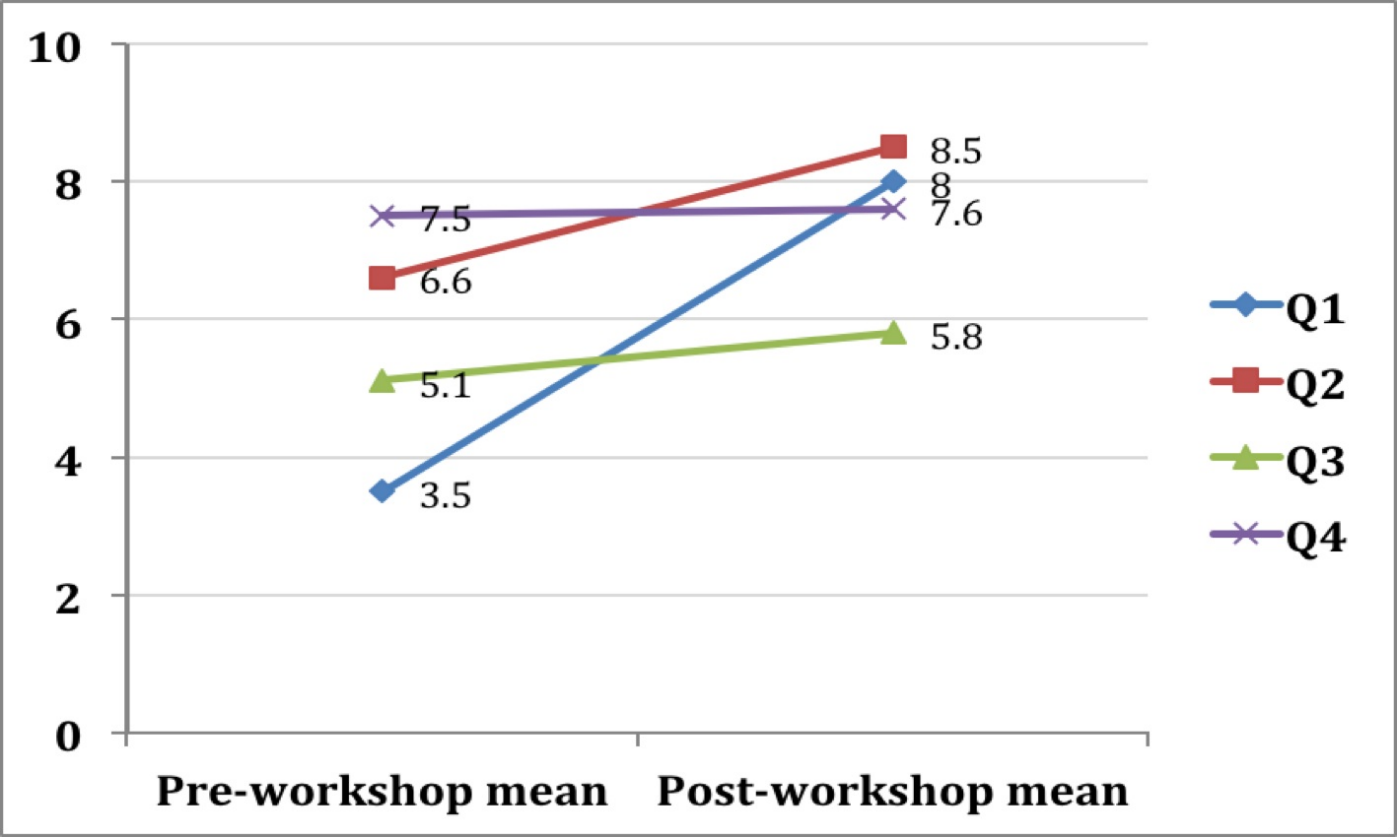
Pre- and post-workshop means for statements #1-4.

One hundred and fifty-four students (100%) completed the voluntary anonymous four statement pre-workshop questionnaire, while 149 students (96.8%) completed the five statement post-workshop questionnaire. Results are depicted in Table 1 and Figure 2.

**Table 1:** Results of the Pre- and Post-workshop Questionnaires Data are mean Likert score/10 (standard deviation) for each statement on the questionnaire.  * - denotes significant result

	Pre-workshop (n = 154)	Post-workshop (n = 149)	P value
Statement #1 “Understanding”	3.5 (2.6)	8 (1.4)	< 0.001*
Statement #2 “Importance”	6.6 (2.2)	8.5 (1.7)	< 0.001*
Statement #3 “Observed poor behaviour”	5.1 (1.9)	5.8 (1.9)	< 0.001 *
Statement #4 “Observed good behaviour”	7.5 (1.5)	7.6 (1.4)	0.6588
Statement #5 “Attention in the future”		7.9 (1.8)	

**Figure 3:**
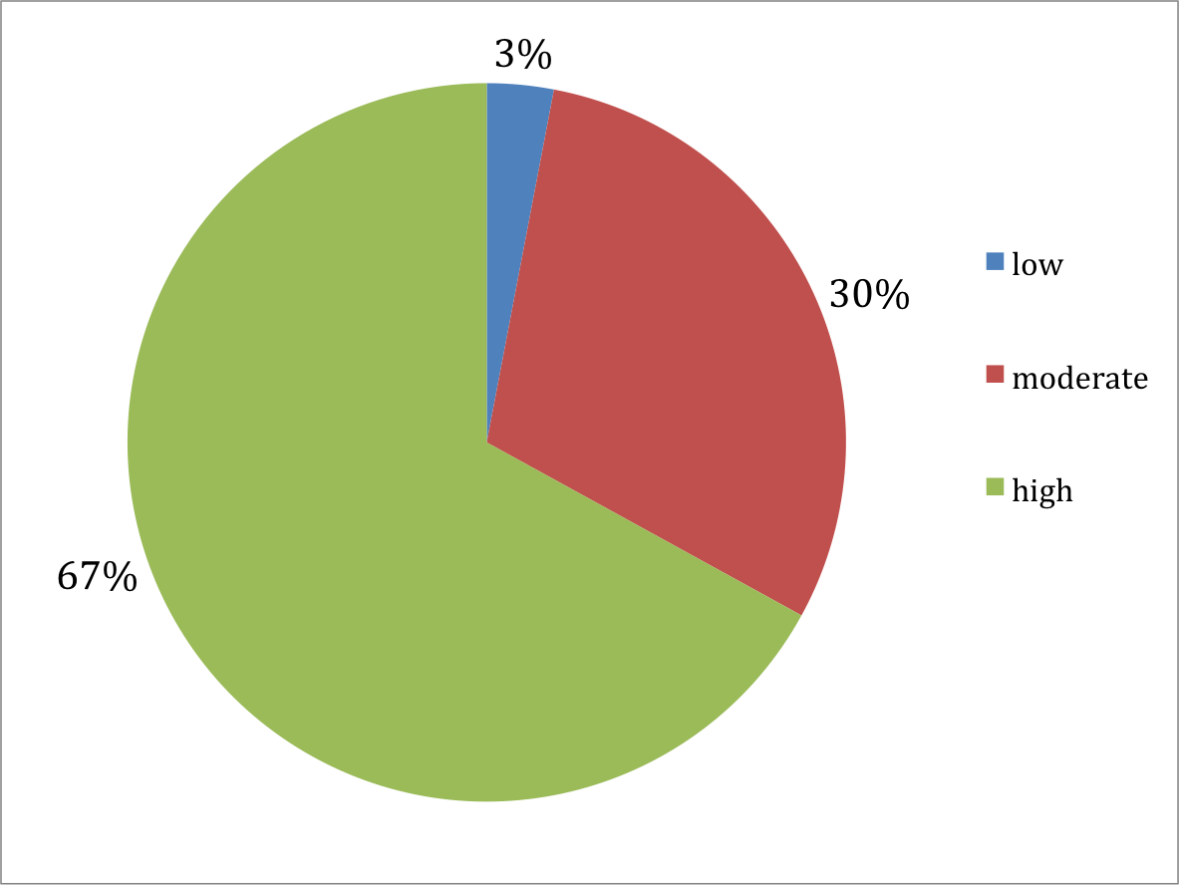
Student grading of the statement "I am likely to pay attention to the hidden curriculum as a result of this lecture" on a 10-point Likert scale, divided into "low," "moderate," and "high" categories. "Low" (grades 0-3), "moderate" (grades 4-7), and "high" (grades 8-10): 2.7%, 30.4%, and 66.9%, respectively.

In the post-workshop questionnaire statement #5, the mean response was 7.9 (SD 1.8). We grouped the responses into three distinct categories, based on how likely the students were to pay attention to the hidden curriculum in the future: 4/148 (2.7%) answered “low" (grades 0-3), 45/148 (30.4%) answered “moderate" (grades 4-7), and 99/148 (66.9%) answered “high" (grades 8-10) (Figure 3).

### Mandatory course evaluation

The mandatory course evaluation yielded 108 distinct voluntary comments about the hidden curriculum workshop, which were grouped into five themes. Each theme is described below, and representative comments are provided. Themes 1-3 refer to a student’s recognition or perception of the concepts of the hidden curriculum (“sensitized”) and knowledge of the entity of the hidden curriculum (“aware”). Table 2 represents the frequencies of comments in each theme.

**Table 2:** Five Themes Derived from Analysis of Voluntary Comments on the Hidden Curriculum Workshop and Frequency of Comments Per Theme

	Responses (N = 108)
1. Not sensitized	8 (7.4%)
2. Sensitized but unaware	12 (11.1%)
3. Sensitized and aware	34 (31.5%)
a. Examples of unprofessional behaviour	10 (29.4%)
b. Responsibility of preceptor for role modelling	8 (23.5%)
c. Acknowledgement of hidden curriculum	16 (47%)
4. Comments on teaching methodologies/environment	43 (39.8%)
5. Suggestions for enhancement	11 (10.2%)

Theme 1: Not Sensitized and Unaware of the Hidden Curriculum

We assigned comments to this theme when the student expressed a lack of recognition of the hidden curriculum or dissatisfaction with the lecture content. These students are possibly the ones that have not come across critical ethical situations and/or do not have the insight to recognize an ethical dilemma. Representative comments included:

“This lecture had little to nothing to do specifically with Ob/Gyn and seems to be in this block only because of the specialty of the presenter.”

“Unnecessary lecture.”

“The lecture went over the designated time which I found was far too long.”

Theme 2: Sensitized but Unaware of the Hidden Curriculum

We assigned comments to this theme when the student alluded to having been confronted with an ethical situation in their clinical experience (e.g., “Very worthwhile lecture – I wish this was discussed prior to my [other specialty] rotation”) but had not realized that the concept of the hidden curriculum existed or had been described (e.g. “I was unaware of the hidden curriculum and found this lecture very beneficial”). Comments in this category, in general, reflected an open attitude and an engagement on the topic. A representative comment is below:

“I feel very honoured to have attended, and I feel more at ease about the impressions I sometimes struggle with as a student – wondering if I am overly sensitive or if there is a larger cultural issue at hand.”

Theme 3: Sensitized and Aware of the Hidden Curriculum

We assigned comments to this theme based on the students’ disclosure of prior exposure to the hidden curriculum or a discourse of their struggle with it (e.g., “The topic is really pertinent and made me think a lot about how conflicted I often find myself in terms of the values I want to hold as a future doctor”). This was the most diverse theme, which we further subdivided into three categories: examples of unprofessional behaviour, the responsibility of the preceptor for role modeling, and the acknowledgment of the hidden curriculum. 

Ten (29.4%) students gave specific poignant examples of unprofessional physician behaviour. One student remarked: “I have witnessed a health care professional saying ‘this patient is nuts’ and ‘our next surgery – big fat lady’. By witnessing this, I felt uncomfortable and I hope that I’ll remember that if I get to teach in the future”.

Interestingly, eight (23.5%) comments questioned the role of the preceptor in modeling appropriate behaviour. Two such comments are given below:

“I think students may feel more comfortable to speak up or act differently if they know the faculty or the clinical [teachers] support them (genuinely) in their denouncement of inappropriate behaviour.”

“It seems like the ‘hidden curriculum’ is a code word for all of the negative behaviours commonly practiced by physicians. I'm not sure if, therefore, the "hidden curriculum" is an appropriate term. I would like to think that few physicians display these types of behaviours and that they are not meant as role model behaviour, which most students are able to realize. Still [the hidden curriculum is] a good thing to discuss so students are aware.”

The largest proportion (47%) of comments in this theme validated the importance of the hidden curriculum teaching, represented by the following:

“I really appreciated this lecture and thought it was quite relevant to our clinical experience. I believe most of us were aware of what the hidden curriculum is as we do experience it on a daily basis. That said, it's always good to get a reminder not to change our practice and not be influenced by those negative mentors.”

Theme 4: Comments on Teaching Methodologies and Environment

We assigned comments to this theme when they reflected on the presentation content, the workshop structure, and the lecturer’s ability to convey information. Students seemed to appreciate a didactic introduction to the topic (“I was impressed by the soundness of the presentation and the use of resources to state its definitions”), group discussion format (“Discussing specific scenarios as an activity during class was very helpful”), and a respectful and engaging attitude of the lecturer (“[Lecturer] is a really engaging lecturer and their enthusiasm and caring for students really comes through”).  

Theme 5: Suggestions for Enhancement  

We assigned comments to this theme when the student comments provided valuable suggestions for improving the workshop. Approximately half of these comments reflected a desire for a longer discussion:

“More time [should be given] for discussing/validating classmates' contributions of their experiences, cases.”

Students also sought guidance on how to react to ethical dilemmas:

“It is not enough to wish that we would stay as we are now, we need specific guidelines and advice on how to manage negative interactions.”

“I think it would be useful to extend the discussion a bit to what can or should we do as medical students or how we can react to different scenarios.”

## Discussion

Two decades of medical education literature on the hidden curriculum have demonstrated it to be an omnipresent, integral, teachable, and evaluative part of medical education. Through a mixed methodology approach, our study contributes to the body of literature by examining the impact of this work within a Canadian context.

Firstly, it seems that despite being exposed and sensitized to the concept of the hidden curriculum in their pre-clerkship years through a longitudinal ePortfolio platform [9], students had a low pre-workshop level of understanding of the hidden curriculum and attributed a lower importance to teaching of the hidden curriculum, as compared to after the workshop. This suggests that a single encounter – such as the workshop – can significantly increase students’ perceived understanding of the subject. It also suggests that ethical concepts can be nurtured by repetition, as demonstrated by the large percentage of students who were more likely to pay attention to the hidden curriculum after the workshop.

The paucity of comments in Theme 1 (Unaware/not sensitized) would suggest that most students have come across an ethical situation and, thus, value opportunities for discussion and reflection with a faculty member and peers.

Secondly, relative indifference in pre- and post-workshop means for statements 3 and 4 suggest that students are already tuned into behaviours demonstrated by their teachers, be they positive or negative. It is encouraging to see that students more frequently recognize encounters with a clinician who modeled positive behaviour. Unfortunately, a large number of comments point to the fact that despite two decades of physician formation focusing on the hidden curriculum, negative attitudes and behaviours are still a part of the medical “lingo”. Nonetheless, an intervention, such as a workshop, can increase awareness and resilience, as the following quote demonstrates: 

“This is a rarely discussed, important topic in medical education. It was timed appropriately following our [other specialty rotation], where each of us encountered the hidden curriculum at some level. Thank you for encouraging us to hold to the values with which we entered medical school.”

Thirdly, a large number of students are not only sensitized and aware of the hidden curriculum but are also armed to handle ethical situations and are recognizing and calling for reform at the resident and faculty level. While a large body of literature addresses various strategies to sensitize medical students to the hidden curriculum, there is a dearth of literature describing similar initiatives for residents, and even less for faculty [16-20].

In reply to the study by Neumann, et al., which found declining levels of empathy as medical students progressed through their clinical training [21], Weissman asked “Could it be that the declines noted in student and resident empathy are caused by their emulating the behaviour of the faculty? Studies are needed to assess faculty empathic levels as contrasted with those of trainees. Without such data, it is difficult or impossible to know if the trainees’ decline in empathic capacity is a learning issue or an issue related to their modeling their interactions with patients on those of the faculty” [22]. Indeed, social theories on learning support that people learn by watching one another via observation, imitation, and modeling [23]. Additionally, studies have noted that students choose medical professions based on mentors [24-25]. Perhaps the most important finding in our study is that a majority of medical students recognize that if the culture of negativity is to change within medicine, it has to come from both sides – the teacher and the learner. It is most poignantly summarized in this student’s comment:

“The culture of medicine impacts students just as much as residents and other learners. It is so bad in fact that it definitely shapes career choice from the very start- as early as a first year 10-hour observership in a specialty is enough to influence a student in his/her career choice decisions. It also sets examples very early (good and bad) about the way physicians treat allied health workers and patients. It appears sometimes the students are more aware of the hidden curriculum than the teachers are. I agree that as a student there are things that can be done to "protect yourself" so to speak - but perhaps we are targeting the wrong side of the equation…”

Future directions include development and implementation of a similar program for residents in Obstetrics and Gynaecology as they are an integral part of the medical team and important in shaping the development of medical students [26]. At an institutional and departmental level, building and actively supporting a culture that models patient-centred care and fosters remediation of unprofessional behaviour is likely to improve the working environment for everyone and minimize any negative influences of the hidden curriculum.

Lastly, the workshop format is an effective and safe milieu for addressing the hidden curriculum within a specialty clerkship rotation. Positive student evaluations of this strategy affirm the previous work of Weissman, et al. [27], which describes individual role-modeling as a primary method of shaping the hidden curriculum. No additional financial or institutional resources were required to address the hidden curriculum, which suggests that it can be initiated and continued with relative ease.

## Conclusions

The hidden curriculum is far from invisible and threatens the inherently caring nature and values that we wish to uphold and protect as physicians. Our study suggests that students are fully aware of its existence and have been “sensitized” through their participation in an interactive and positively evaluated workshop. Strengths of our study include a > 95% response rate to two surveys, a large number of participants, and standardized delivery of workshop material by the same lecturer. Drawbacks include the subjective nature of grading scales and recall bias. Some of the observed measures may be reflective of progressive clinical experiences.

What we are like as physicians is just as important as what we know. Addressing the hidden curriculum is important to the development of medical students, and it is hoped that the next generation of physicians will be much less blind to its existence. The workshop presented to students at the University of Ottawa could be easily shared with other medical schools and facilitated by any physician. It is likely that recognition and awareness of the hidden curriculum will be just as rejuvenating for the faculty as for the students.
